# QSAR Studying of Oxidation Behavior of Benzoxazines as an Important Pharmaceutical Property

**Published:** 2017

**Authors:** Elham Baher, Naser Darzi

**Affiliations:** a *Faculty of science, Department of chemistry, Golestan University, Gorgan, Iran. *; b *Faculty of science, Department of chemistry, Azad University of Mashad, Mashad, Iran.*

**Keywords:** Benzoxazines, Half wave potential, Artificial neural network, pharmaceutical property, Quantitative structure-property relationship

## Abstract

In this work the electrooxidation half-wave potentials of some Benzoxazines were predicted from their structural molecular descriptors by using quantitative structure-property relationship (QSAR) approaches. The dataset consist the half-wave potential of 40 benzoxazine derivatives which were obtained by DC-polarography. Descriptors which were selected by stepwise multiple selection procedure are: HOMO energy, partial positive surface area, maximum valency of carbon atom, relative number of hydrogen atoms and maximum electrophilic reaction index for nitrogen atom. These descriptors were used for development of multiple linear regression (MLR) and artificial neural network (ANN) models. The statistical parameters of MLR model are standard errors of 0.016 and 0.018 for training and test sets, respectively. Also, these values are 0.012 and 0.017 for training and test sets of ANN model, respectively. The predictive power of these models was further examined by leave-eight-out cross validation procedure. The obtained statistical parameters are Q^2 ^= 0.920 and SPRESS = 0.020 for MLR model and Q^2 ^= 0.949 and SPRESS = 0.015 for ANN model, which reveals the superiority of ANN over MLR model. Moreover, the results of sensitivity analysis on ANN model indicate that the order of importance of descriptors is: Relative number of H atom > HOMO energy > Maximum electrophyl reaction index for N atom > Partial positive surface area (order-3) > maximum valency of C atom.

## Introduction

Benzoxazine is the reaction product of amine, phenol and formaldehyde which can provide polymers with high glass transition temperature, low water absorption, excellent physical and electrical performances and excellent fire resistance properties (1). One of the interesting features of these compounds is the drug properties. These were used as bacteriocides, fungicides, antitumor agents, herbicides, microbiocides or anti-inflammatory agents, tyrosine mimetics, bacteriostatic, immunomodulating agents, neuroprotective antioxidants and were used as antituberculotic agents in various cancer chemotherapy regimens for the treatment of sarcoma and cerebral tumors. (2-10). 

Oxidation reactions played an important role in establishing structures and chemical properties of benzoxazines. Since these reactions are the most common pathways at the first phase of drug biotransformation. Therefore the electrochemical half wave potential of benzoxazines can be directly useful for investigation of their biological properties (11). Drugs with low oxidation potential can operate more successfully in the treatment of disease. A common technique in studies of electro-oxidations of benzoxazines is the voltammetric method (12). This method is the electrochemical technique. Since generation and examination of new drugs based on benzoxazine and its investigation by the voltammetric method is limited in time and cost (13) therefore, the development of theoretical model to predict the properties of these compounds are interesting and necessary. Quantitative structure activity relationship (QSAR) methods enable in prediction and interpretation of the properties and activities of a wide range of drugs and organic compounds based on the correlation between their properties and molecular characteristics (molecular descriptors) (14-18). There are some reports about the applications of QSPR/QSAR in electrochemistry (19-22). Wei *et al.* studied the relationship between the reduction characteristics and molecular structure of 87 chlorinated aromatics, such as naphthalenes, biphenyls, benzenes and phenols (19). Shamsipur and Hemmateenejad employed principle component regression (PCR) and principle component artificial neural network (PC-ANN) models in QSPR study of E_1/2 _of some organic compounds. Their best PC- ANN model can explain the 96% of variances in the E1/2 data (22). Fatemi *et al.* applied support vector machine (SVM) for the prediction of selectivity coefficients of anion-selective electrode for some univalent anions. The calculated root-mean-square errors of SVM for training and test set of their model are: 0.878 and 0.890 respectively. The correlation coefficients of training and test set are: 0.95 and 0.94, respectively. Also the obtained statistical parameters of cross-validation test on SVM model were: *Q*^2^ = 0.858 and SPRESS = 1.050, which revealed the reliability of their model (23). Gallegos *et-al*. developed some models to predict the logarithm of minimum inhibitory concentration (log MIC) of a subset of 39 substituted benzoxazines using a quantum molecular similarity approach (24). Nesmerak *et al*. was used Hammet substitutent constants (25) for calculation of half-wave potentials for 40 benzoxazine (20). Toropov *et-al.* calculated optimal descriptors with simplified molecular input line entry system (SMILES) notation of same Nesmerek group chemicals (26) and used QSPR modeling to calculate the electrochemical half-wave potentials of these compound (26). 

In the present work, we tried to predict the half-wave potential of some benzoxazine derives from their molecular descriptors by using artificial neural network (ANN) and multiple linear regression (MLR) techniques. 

## Experimental

*Software*

The 3D structures of the studied compounds were optimized using semi-empirical quantum-chemical methods of AM1 in HyperChem (Ver.7) package (27). The structural descriptors are numerical values that encode structural features of the molecular structures. In the present work, the CODESSA software was used to calculate 393 constitutional, topological and geometrical descriptors (28). Then heuristic method (HM) was used to search the best set of descriptors for multilinear correlations (29). 

Artificial neural networks are mathematical systems that simulate biological neural networks (30-32). They consist of processing elements (nodes or neurons) which organized in some layers. Back-propagation neural networks are most often used in analytical applications. The back-propagation network receives a set of inputs, which are multiplied by each node and then a nonlinear transfer function is applied for their processing. The goal of training the network is to change the weights between the layers in a direction to minimize the output errors. More details about the theory of the neural networks have been adequately described in many literatures (32-38). The ANN programs were written in FORTRAN 77 in our laboratory. A three-layer network with a sigmoid transfer function was design for each ANN. The generated artificial neural network uses descriptors selected by HM as inputs. The number of nodes in the input layer is dependent on the number of descriptors introduced in the network. The number of nodes in the output layer for both subsets (training and test sets) was set to be one. The inputs and outputs values of ANN were normalized between 0.1 and 0.9. The initial weights were selected randomly between −0.3 and 0.3. The number of nodes in the hidden layers, learning rate, and momentum would be optimized before training the network. During the training of ANN, the values of weights and biases continuously changed to minimize the differences between ANN outputs and desired activity/property values, using the back propagation of errors. In order to evaluate the performance of the ANN, the standard error of training (SET) and the standard error of prediction (SEP) were used. The training iteration was stopped at overtraining point, where SEP is started to increase. Then the trained network was used to calculation the E_1/2_ values of test set. In order to further investigation of the credibility of obtained ANN model leave-eight-out cross validation method was used. Finally, the sequential zeroing weight (SZW) approach was used for evaluation of the relative importance of selected molecular descriptors.

*Dataset and Molecular descriptors*

**Figure 1:**
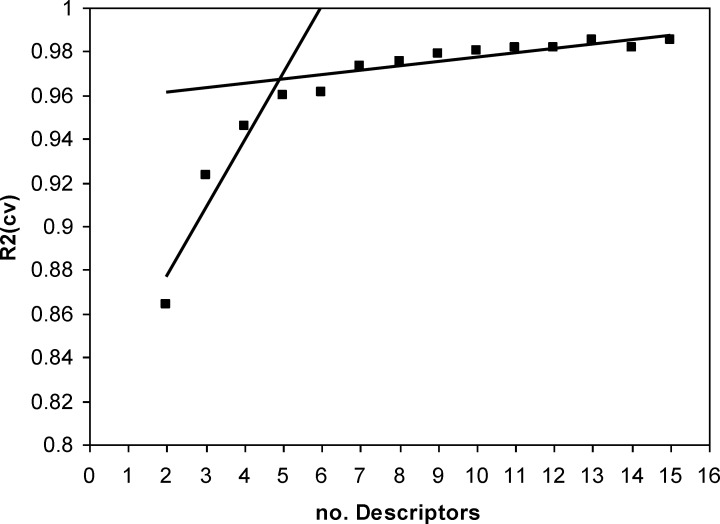
Plot of *R*2 for the obtained models versus the number of descriptors involved

New groups of antimycobacterial agents that were studied in the present work as dataset are derivatives of benzoxazine which their half-wave potentials were obtained from reference (20). The electrochemical measurements on these compounds were performed by an EKO-Tribo Polarograph. The reference electrode was a silver plate which immersed in a solution of acetonitrile that consists 0.01M of AgNO_3_ and 1M of NaClO_4_. The chemical structures and experimental oxidation half-wave potential of these compounds are shown in Figure 1 and Table 1. The 3D structures of the studied compounds were optimized using semi-empirical quantum-chemical methods of AM1. The data set was separated into two groups: training and test sets. All molecules were placed by Y-ranking method in these sets. The training set, consisted of 35 molecules, was used for the model generation and the test set, consisted of 5 molecules, was used to take care of the overtraining and evaluate the prediction power of the generated model.

*Data screening and descriptor selection*

The CODESSA software was used to calculate constitutional, topological and geometrical descriptors. Then heuristic method (HM) was used to search the best set of descriptors for multilinear correlations. In the first step of this method, descriptors with constant values for all molecules were eliminated from the pool of descriptors. Also, pairs of variables with a correlation coefficient greater than 0.90 were classified as intercorrelated variables, and only one of them was used in developing the model. In deleting one descriptor from one pair of correlated descriptors we tried to keep descriptor which has these criteria: 1) has the higher correlation with independent variable (half wave oxidation potential), 2) its calculation is simpler and easier, 3) has more information about interested activity/properties, 4) is more interpretable. At the end of this step total of 284 descriptors were reminded to further investigations. 

A major decision in developing successive QSPR model is when to stop adding descriptors to the model during the forward selection procedure. A simple technique to control the model expansion is the ‘break-point’ procedure (39). In this method, improvement of the statistical quality of the models is analyzed by plotting the squared correlation coefficient values (R^2^) of the obtained models versus the number of descriptors involved in each model. Consequently, the model corresponding to the break point is considered as the best/optimum model. 

**Figure 2:**
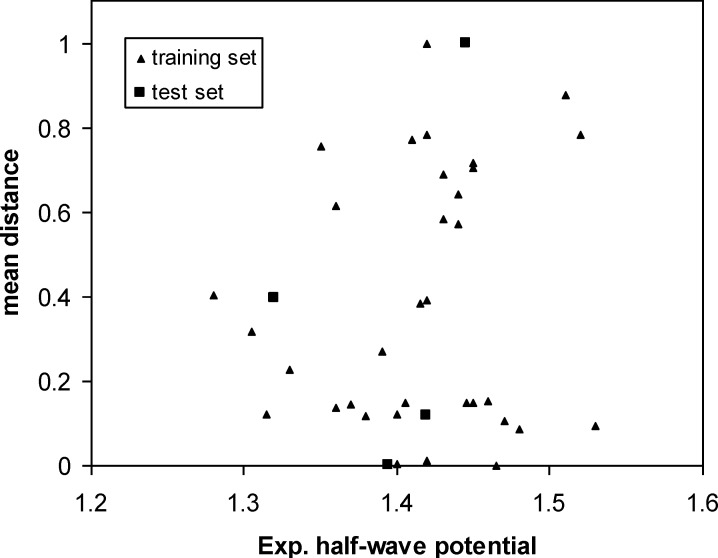
Scatter plot of samples for training and test sets according to the mean distances distribution

Thus, HM procedure was applied to the training set and multilinear regression equations of up to 16 descriptors were developed. Variations of R^2^ against the number of descriptors in the models were recorded and are shown in Figure 2. The application of the break-point algorithm led to the conclusion that the best model had five parameters. The specifications of this model w shown in Table 2. Then the artificial network was used to calculation the E_1/2_ values of training and test set, respectively. Also, in order to further investigation of the credibility of obtained ANN model leave-8-out cross validation method was used. Finally, the sequential zeroing weight (SZW) approach was used for evaluation of the relative importance of selected molecular descriptors.

## Result and discussion

*Molecular diversity validation*

Diversity is a fundamental research subject in chemical database analysis of sampling (40). Molecular diversity analysis explores the way of molecules to cover a determined structural space and underlies many approaches for compound selection and design of combinatorial libraries. The diversity problem involves defining a diverse subset of “representative” compounds so that researchers can scan only a subset of the huge database each time. Therefore, the choice of an optimal metric space that represents the structural diversity of a compound population is determinant in the efficiency of the model (38, 41). In this work, diversity analysis was done for the data set to make sure the structures of the training or test sets could represent those of the whole ones.

For a database of n compounds generated from m highly correlated chemical descriptors, a distance score *(*d_ij_*) *for two compounds *X*_i_ and *X *_j_ can be measured by the Euclidean distance norm based on the compounds descriptors: 

 (1) $$d_{\mathrm{ij}}=\left\|X_{i}\right.-\left.X_{j}\right\|=\sqrt[{2}]{\sum_{k=1}^{m}{\left(x_{\mathrm{ik}}-x_{\mathrm{jk}}\right)}^{2}}$$

Each compound *X*_i_ is represented as a vector:

*Xi *= *(x*_i__1_*, x*_i__2_*, x*_i__3_*, . . . , x*_im_*)*^T ^ for i = 1*, *2*, . . . , *n

where x_i j_ denotes the value of descriptor j of compound X_i_ and T indicates vector transposition. The mean distances ( ) of one sample to the remaining ones were computed as follow:

${\overset{\text{̅}}{d}}_{\mathrm{ij}}=\frac{\sum_{j=1}^{n}d_{\mathrm{ij}}}{n-1}$

(2)

**Figure 3:**
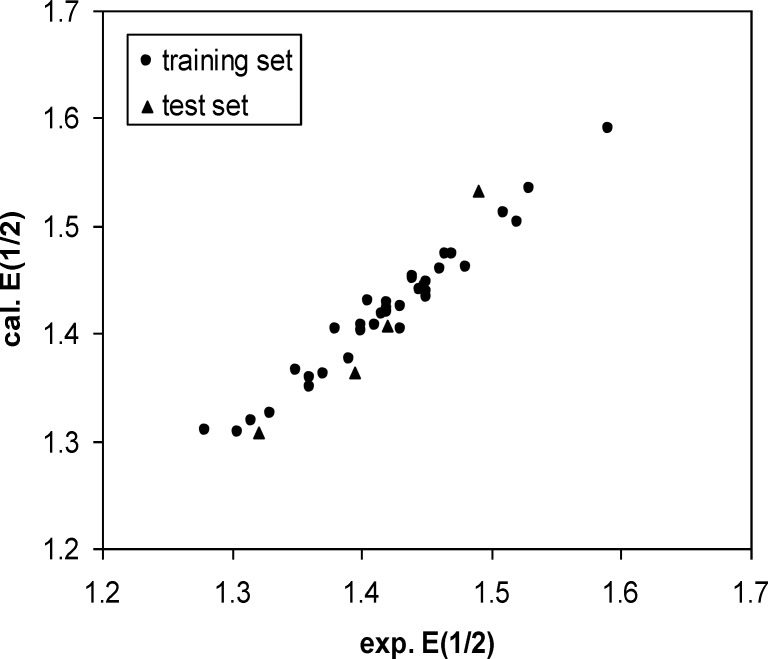
Calculated. E_1/2_ versus Experimental E1/2 plot

Then the mean distances were normalized within the interval (0*, *1). In our data sets, the mean distances of samples versus oxidation half-wave potential are plotted in Figure 3. The distribution of points in this figure illustrates the diversity of the molecules in the training and test sets. As can be seen from this figure, the structures of compounds are diverse in the training and test sets. The training set with a broad representation was adequate to ensure model stability. 

**Table 1:** Structures, experimental, MLR and ANN-predicted values of oxidation half-wave

**Derivate**	**X**	**R1**	**R2**	**E**_(1/2)_ **-Exp**	**E**_(1/2)_ **-MLR**	**E**_(1/2)_ **-ANN**
1	O	7-OCH_3_		1.420	1.413	1.419
2	O	7-OCH_3_	4-F	1.430	1.434	1.424
3	O	7-OCH_3_	4-Br	1.440	1.465	1.453
4	O	7-OCH_3_	3-F	1.445	1.458	1.441
5	O	7-OCH_3_	3-Cl	1.450	1.461	1.447
6	O	7-CH_3_	4-CH_3_	1.415	1.412	1.418
7	O	6-CH_3_	4-CH_3_	1.420	1.416	1.423
8^T^	O		4-Br	1.490	1.522	1.533
9	O	6-OCH_3_	4-CH_3_	1.450	1.425	1.433
10	O	6-OCH_3_	4-F	1.460	1.480	1.460
11	O	6-OCH_3_	4-Br	1.465	1.487	1.474
12	O	6-OCH_3_	4-Cl	1.470	1.487	1.473
13	O	6-OCH_3_	3-F	1.480	1.481	1.462
14	O	6-OCH_3_	4-CN	1.510	1.516	1.512
15	O	6-Cl		1.530	1.525	1.535
16	O	6-Cl	3-Cl	1.590	1.589	1.591
17	S	7-OCH_3_	4-CH_3_	1.280	1.312	1.309
18	S	7-OCH_3_		1.315	1.323	1.318
19	S	7-OCH_3_	4-F	1.350	1.348	1.366
20	S	7-OCH_3_	4-Br	1.360	1.378	1.359
21	S	7-OCH_3_	4-Cl	1.370	1.368	1.362
22	S	7-OCH_3_	3-F	1.390	1.363	1.376
23^T^	S	7-OCH_3_	3-Cl	1.395	1.370	1.364
24	S	7-OCH_3_	4-CF_3_	1.405	1.433	1.430
25^T^	S	7-OCH_3_	3,4-Cl_2_	1.420	1.417	1.408
26	S	7-CH_3_	4-CH_3_	1.305	1.323	1.308
27^T^	S	6-CH_3_	4-CH_3_	1.320	1.328	1.308
28	S		4-Br	1.420	1.449	1.428
29	S	6-OCH_3_	4-CH_3_	1.330	1.336	1.326
30	S	6-OCH_3_		1.360	1.353	1.350
31	S	6-OCH_3_	4-F	1.380	1.395	1.403
32	S	6-OCH_3_	4-Br	1.400	1.406	1.408
33	S	6-OCH_3_	4-Cl	1.400	1.402	1.402
34	S	6-OCH_3_	3-F	1.410	1.399	1.407
35	S	6-OCH_3_	3-Cl	1.430	1.405	1.404
36	S	6-OCH_3_	4-CF_3_	1.440	1.455	1.451
37^T^	S	6-OCH_3_	3,4-Cl_2_	1.445	1.451	1.444
38	S	6-OCH_3_	4-CN	1.450	1.437	1.438
39	S	6-Cl		1.420	1.443	1.420
40	S	6-Cl	3-Cl	1.520	1.498	1.503

T: denotes the test set.

**Table 2:** Specification of multiple linear regression model

**Name of descriptors**	**Symbol**	**Coefficient**	**SE**	**Mean effect**
Relative number of H atom	X_1_	-0.13	±0.027	-0.796
Partial positive surface area(order-3)	X_2_	-0.1	±0.004	-0.086
Maximum electrophyl reaction index for N atom	X_3_	0.023	±0.002	0.075
HOMO energy	X_4_	-0.079	±0.051	1.010
Maximum valency of C atom	X_5_	2.298	±1.012	8.880
Constant		-7.903	±3.65	

n =35, R =0.97, SE = 0.016, F =512

**Table 3:** Internal correlation matrix between molecular descriptors

	**X**_1_	**X**_2_	**X**_3_	**X**_4_	**X**_5_
X_1_	1.000	0.255	0.075	0.650	0.670
X_2_		1.000	-0.027	-0.010	-0.163
X_3_			1.000	-0.352	0.009
X_4_				1.000	0.253
X_5_					1.000

**Table 4:** The statistical results of ANN and MLR models

**Models **	**Training set**		**Test set**		**Cross-validation Test**	
	R	SE	R	SE	Q^2^	SPRESS
ANN	0.983	0.012	0.971	0.017	0.949	0.015
MLR	0.969	0.016	0.970	0.018	0.920	0.020

R, SE, Q^2^ and SPRESS are regression coefficient, standard error, correlation coefficient of cross validation and square of predictive error sum of squares respectively.

**Table 5:** Architecture of ANN

**Transfer Function**	**Sigmoidal**
No. of Hidden Layer Nods	2
Weight Learning Rate	0.2
Bias Learning Rate	0.6
Momentum	0.3
No. of Input Layer Nods	5
No. of Output Layer Nods	1

**Figure 4:**
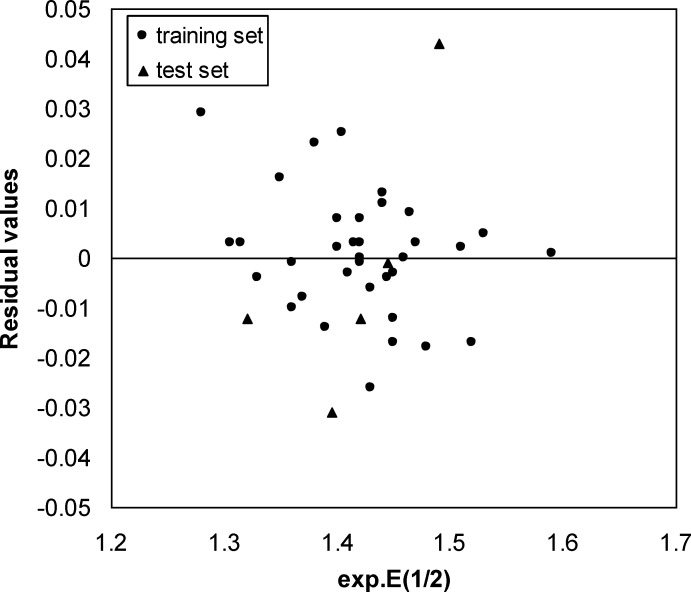
Residual versus Experimental E1/2 plot

**Figure 5:**
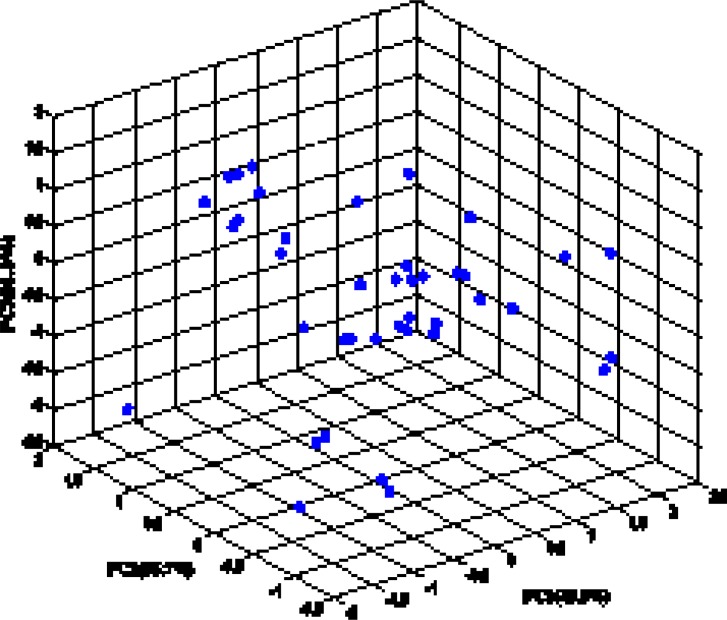
Principal component analysis on the selected molecular descriptors for the consensus model

**Figure 6:**
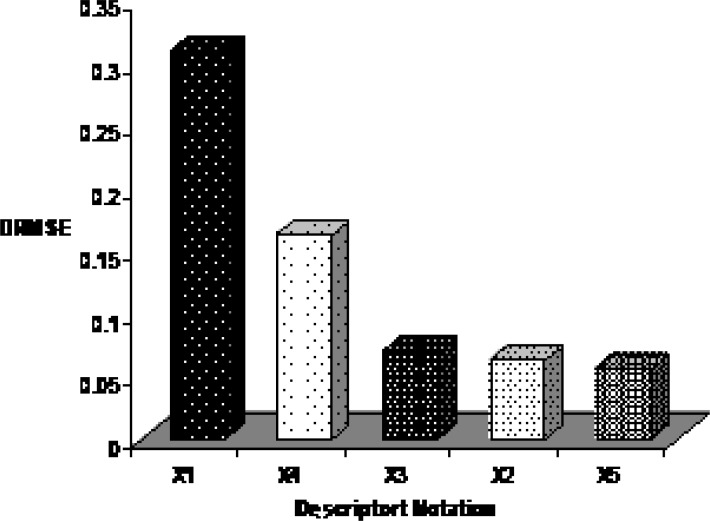
Sensitivity analysis results

*Linear modeling*

The SPSS software (Ver. 14) was used to developing many MLR models (43). The best model was selected based on the statistics of correlation coefficient (R), standard error (SE) and Fisher-statistics value (F). Consequently, among different models, the five-parameter model was chosen based on the break point procedure. Descriptors which were selected by this method are: high occupied molecular orbital energy(HOMO), partial positive surface area, maximum valency of carbon atom, relative number of hydrogen atoms and maximum electrophilic reaction index for nitrogen atom that have shown in Table 2. 

Multicollinearity for the selected parameters (descriptors) was also checked and its result was presented in Table 3. As can be seen in this table there are not any high correlation between these descriptors. Then the MLR model was used to calculate of E_1/2_ for test set as well as training set. The MLR predicted values of E_1/2_ were shown in Table 1. Finally, the leave 8-out cross-validation (L8O) was used to evaluate credibility and robustness of these models. The statistical parameters of this test were shown in Table 4. Other statistical parameters of MLR model are: average error = 0.0002, relative error = 0.0022 and absolute error = 0.0102, respectively.

*Non-linear modeling*

A three-layer network with a sigmoid transfer function was designed for ANN model. The network was trained using the training set by the back propagation strategy for optimization of the weights and bias values. To obtain the best result the weight and bias learning rate and momentum value as well as ANN’s topology were optimized. The procedure for optimization of ANN’s parameters is given elsewhere (37, 38). The optimized values of these terms and ANN characteristics are given in Table 5. Then the constructed ANN model was used to calculate the E_1/2_ for test set as well as training set. The predicted values of E_1/2_ by ANN model were shown in Table 1. Moreover, the leave-8-out cross-validation (L8O) was used to evaluate the credibility and robustness of the ANN model. The statistical parameters of this test were shown in Table 4. Other statistical parameters of ANN model are, average error = 0.0046, relative error = 0.0040 and absolute error = 0.0137, respectively. In comparison whit MLR statistical parameters and other statistical values in Table 4, it can be seem that the performance of ANN model was better than MLR ones. Figure 4 indicates the variation of ANN predicted against experimental values of E_1/2_ that the agreement between the predicted and experimental values is clear (R _(training set) _=0.0983 and R _(test set) _=0.971). Also, the residual values between ANN predicted and experimental values of half-wave electrooxidation potential of benzoxazines were traced in Figure 5. 

The random distribution of residuals about zero line confirms that there is no systematically error in developed ANN model. To verify the chemical domain of the consensus model and the distribution of the studied chemicals in this new multidimensional space, the chemicals are plotted in a principal components 3D-graph (Figure 6), which was obtained by applying PCA on all molecular descriptors used by these models. This PCA plot shows that chemicals have fine distribution in the molecular descriptors domain.

*Sensitivity analysis and descriptor interpretation*

By interpreting selected descriptors in the ANN model, it is possible to gain some insight into the factors that are likely to govern the E_1/2_ of benzoxazines. Here, a brief interpretation of these factors in order to determine the relative importance of each variable is given based on the results of sensitivity analysis. The procedure of this approach is based on the sequential removal of variables by zeroing the specific connection weights for that specific input variable in the first layer of the ANN (44). For each sequentially zeroed input variable, root mean square error of prediction set (RMSEP) as the prediction error of this network was calculated. Generally RMSEP value increases in this way. Then, differences between RMSEP and root mean square error of established ANN (RMSE) was calculated and shown as DRMSE. Each variable which causes greater value of DRMSE is more important. The DRMSE values are shown for each descriptor in Figure 7. As the mentioned earlier, five descriptors were used for ANN model to comprise: relative number of H atom, HOMO energy, maximum electrophyl reaction index for N atom, partial positive surface area (order-3), maximum valency of C atom that belonging for constitutional, quantum chemical and charge descriptors and encode electronic aspects of the molecular structure. The order of importance of descriptors is: Relative number of H atom > HOMO energy > Maximum electrophyl reaction index for N atom > Partial positive surface area (order-3) > maximum valency of C atom.

First important descriptor in the model is relative number of H atom that is a simple constitutional type descriptor. This factor indicates the size of molecules as well as the degree of saturation of molecule. The second one is the highest occupied molecular orbital energy which is belonging to quantum chemical descriptors and determines the needed energy to drawing the electron in oxidation process (45). Molecule with high HOMO energy values can donate its electron more easily than the molecule with lower HOMO value, and hence is more reactive (26). Next descriptor is maximum electrophylic reaction index for N atom that is the quantum chemical descriptor too. This index provides feasible chemical interaction with electrophilic attack as electron affinity (46) and is important in molecular properties and reactivity in particular for radical reactions. 

The forth descriptor is partial positive surface area (order-3) which is a charge descriptor and contains the electronic and structural information of molecule (46). This descriptor encodes the solvent accessible surface area of molecule in electrochemical reaction, and can well estimate the absolute hardness and can affect on electrooxidation of benzoxazines. The last descriptor is maximum valency of C atom. This parameter is a charge type descriptor which can affect on electron affinity of the molecule and therefore can correlate to the E_1/2_ of a molecule. Thus these descriptors can encode different aspects of molecules which can effect on their E_1/2_ values.

## Conclusion

The obtained results indicate that QSPR approaches can be used to predict the electrooxidation half-wave potentials of benzoxazine derivatives from their structural molecular descriptors. Also, comparison between statistical parameters of ANN and MLR models indicates that the ANN model produces better results due to non-linear characteristic of ANN. Finally, total descriptors which were appeared in this model can encode features of molecules which were responsible in electrooxidation characteristics of these molecules. 
